# Surgical explantation for a rapid deployment valve: the ‘shave-and-hook technique’

**DOI:** 10.1093/icvts/ivae072

**Published:** 2024-04-18

**Authors:** Kyokun Uehara, Taku Shirakami, Yoshio Arai

**Affiliations:** Department of Cardiovascular Surgery, Tenri Hospital, Nara, Japan; Department of Cardiovascular Surgery, Tenri Hospital, Nara, Japan; Department of Cardiovascular Surgery, Tenri Hospital, Nara, Japan

**Keywords:** Rapid deployment valve, Explantation, Aortic valve replacement

## Abstract

An 80-year-old female patient underwent redo aortic valve replacement for haemolysis caused by moderate paravalvular leakage 1 year after a 21-mm Intuity Elite valve implantation. The elevatorium passed at the segment with paravalvular leakage. The frame was then bent inward using a hook and the peel around the sawing ring was shaved by an elevatorium. After explantation of the Intuity Elite valve, endoscopic examination showed no sign of annular or sub-annular damage. Conventional aortic valve replacement using a biological valve was performed. We introduce a safe alternative technique for explantation of a rapid deployed valve.

## INTRODUCTION

Recently, a sutureless valve (Perceval; Livanova PLC, London, UK) and rapid deployment valve (Intuity Elite; Edwards Lifesciences, Irvine, CA, USA) have been widely implanted during aortic valve replacement (AVR) [1, 2]. These valves have offered an alternative to conventional AVR with 0.7–2.5% of 30-day mortality, but surgical explantation was required in 3% of all cases during follow-up [1, 2]. Surgical techniques of explantation have been reported only after Perceval implantation [3, 4]. We report herein a ‘shave-and-hook’ technique for the explantation of an Intuity Elite valve implanted 1 year earlier. 

## PATIENT

An 80-year-old female patient was referred to our department with haemolysis. She had undergone AVR via median full sternotomy using a 21-mm Intuity Elite valve one year earlier. During AVR, additional stitches had been applied to close minor paravalvular leakage (PVL) at the commissure of left and non-coronary sinuses of Valsalva. Lactate dehydrogenase concentration increased to 1700 U/l and total bilirubin reached 1.6 µg/dl and transoesophageal echocardiography revealed moderate PVL at the commissure between the left and right sinuses of Valsalva. Redo conventional AVR was planned for haemolysis.

## SURGICAL TECHNIQUE

**Figure 1:**
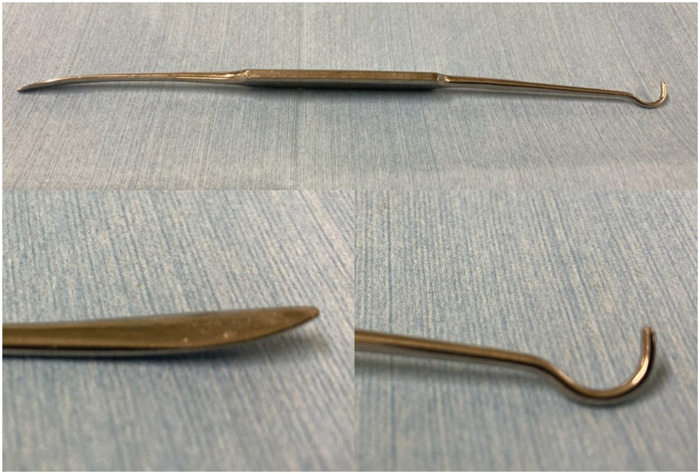
Elevatorium and hook.

**Figure 2:**
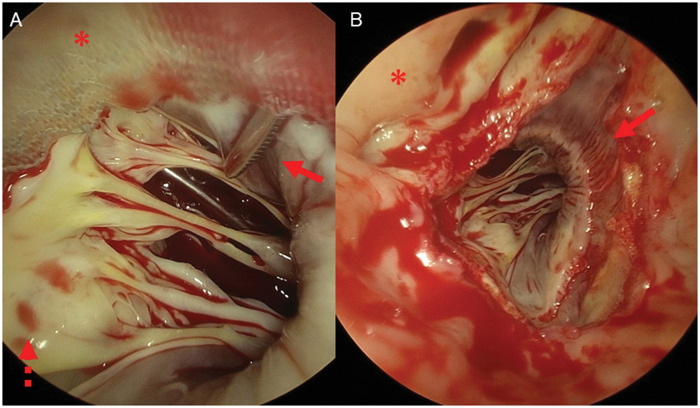
(**A**) Endoscopic examination confirmed the elevatorium passing through the gap to the left ventricle (solid arrow). Shielding cloth of the Intuity Elite valve (asterisk), and the mitral valve (dot arrow). (**B**) Endoscopic examination after explantation of the Intuity Elite valve. Annular and sub-annular structures were preserved (arrow). The left coronary artery ostium (asterisk).

Redo AVR was performed through re-median sternotomy. After opening the ascending aorta, the segment with PVL was found at the commissure between the left and right sinuses of Valsalva, and endoscopic examination confirmed passage of the elevatorium (elevatorium and hook; Mizuho, Tokyo, Japan) (Fig. 1) through the gap to the left ventricle (Fig. 2A). Three guiding sutures at each nadir on the sawing ring, and additional stitches to close the minor PVL were detached. Blunt dissection was performed between the native aortic annulus and the sawing ring of the Intuity Elite valve. The peel around the sawing ring was easily shaved using the elevatorium. Opening the bovine pericardial leaflet, the frame was carefully investigated at the left ventricular outflow tract (LVOT). The frame at the segment showing PVL was then bent inward using the hook. In this case, the frame on the annulus of the left cusp was bent first. The frame on the non-coronary side was then bent inward using the hook. The Kelly forceps grasped the stent post of the commissure between the right and left sinuses of Valsalva to gently lift the valve. After carefully separating the frame and LVOT in the right sinus of Valsalva using the elevatorium, the Intuity Elite valve was explanted (Video 1). Endoscopic examination showed no sign of annular or sub-annular damage (Fig. 2B). Conventional AVR was performed using a 21-mm INSPIRIS valve (Edwards Lifesciences, Irvine, CA, USA) in a supra-annular position. Aortic cross-clamping time was 89 min. Transoesophageal echocardiography showed no PVL. The pathological examination showed no sign of infection on the explanted valve. The patient was transferred to a rehabilitation hospital on postoperative day 15 without any sign of heart failure.

## COMMENTS

Surgical explantation after a sutureless valve or rapid deployment valve implantation was required in 3% of all cases [1, 2], and surgical techniques of explantation have been reported after Perceval implantation [3, 4]. The Intuity Elite valve consists of a balloon-expandable cobalt-chromium alloy frame covered by shielding cloth made of a knitted polyester fabric. After the prosthesis is positioned, the balloon expands the valve frame within the LVOT, establishing a seal between the aortic annulus and frame. Therefore, in cases of device retrieval, one concern is damage to sub-annular structures, including the mitral valve and membranous septum. Carefully bending the frame inward at the two sinuses of Valsalva facilitates valve removal. Endoscopic examination helps in the evaluation of the LVOT, and the hook can easily grasp the frame tightly. A small nerve hook may be too weak to bend the frame of cobalt-chromium alloy. In our case, redo AVR was performed 1 year after the Intuity Elite valve implantation, so dissection between the native aortic annulus and sawing ring of the Intuity Elite valve could be performed simply by shaving with the elevatorium. Considering additional stiches for minor PVL during the Intuity implantation and the PVL at the different segment during the explantation, PVL was attributed to inadequate balloon inflation pressure or time to deploy the stent frame. A larger valve size might be better for future transcatheter AVR, however, considering the patient’s age, the 21-mm valve was selected without annulus enlargement. The shave-and-hook technique might be an alternative for explantation of the Intuity Elite valve and endoscopic examination is beneficial to evaluate sub-annular damage during reoperation.

**Conflict of interest:** none declared.

## Data Availability

The data underlying this article will be shared on reasonable request to the corresponding author.
